# Performance of GPT-5 Frontier Models in Ophthalmology Question Answering

**DOI:** 10.1016/j.xops.2025.101034

**Published:** 2025-12-06

**Authors:** Fares Antaki, David Mikhail, Daniel Milad, Danny A. Mammo, Sumit Sharma, Sunil K. Srivastava, Bing Yu Chen, Samir Touma, Mertcan Sevgi, Jonathan El-Khoury, Pearse A. Keane, Qingyu Chen, Yih Chung Tham, Renaud Duval

**Affiliations:** 1 Cole Eye Institute, Cleveland Clinic, Cleveland, Ohio; 2 Department of Ophthalmology, University of Montreal, Montreal, Quebec, Canada; 3 Department of Ophthalmology, Centre Hospitalier de l'Universite de Montreal, Montreal, Quebec, Canada; 4 The CHUM School of Artificial Intelligence in Healthcare (SAIH), Centre Hospitalier de l'Université de Montréal (CHUM), Montreal, Quebec, Canada; 5 Temerty Faculty of Medicine, University of Toronto, Toronto, Ontario, Canada; 6 Department of Ophthalmology, Hopital Maisonneuve-Rosemont, Montreal, Quebec, Canada; 7 Cleveland Clinic Lerner College of Medicine of Case Western Reserve University, Cleveland, Ohio; 8 Neurological Institute, Cleveland Clinic, Cleveland, Ohio; 9 Institute of Ophthalmology, University College London, London, UK; 10 NIHR Biomedical Research Centre at Moorfields, Moorfields Eye Hospital NHS Foundation Trust, London, UK; 11 Department of Biomedical Informatics and Data Science, Yale School of Medicine, Yale University, New Haven, Connecticut; 12 Department of Ophthalmology, Centre for Innovation and Precision Eye Health, Yong Loo Lin School of Medicine, National University of Singapore, Singapore; 13 Singapore Eye Research Institute, Singapore National Eye Centre, Singapore; 14 Eye Academic Clinical Program, Duke NUS Medical School, Singapore

**Keywords:** Artificial intelligence, Foundation models, GPT-5, Large language models, Ophthalmology

## Abstract

**Purpose:**

Novel large language models (LLMs) such as Generative Pretrained Transformer-5 (GPT-5) integrate advanced reasoning capabilities that may enhance performance on complex medical question-answering tasks. For this latest generation of reasoning models, the configurations that maximize both accuracy and cost-efficiency have yet to be established. Our objective was to evaluate the performance and cost-accuracy trade-offs of OpenAI’s GPT-5 compared with previous generation LLMs on ophthalmic question answering.

**Design:**

Evaluation of diagnostic test or technology.

**Participants:**

Generative Pretrained Transformer-5 is a publicly available LLM.

**Methods:**

In August 2025, 12 configurations of OpenAI’s GPT-5 series (3 model tiers across 4 reasoning effort settings) were evaluated alongside o1-high, o3-high, and GPT-4o, using 260 closed-access multiple-choice questions from the American Academy of Ophthalmology Basic Clinical Science Course data set. The study did not include human participants.

**Main Outcome Measures:**

The primary outcome was accuracy on the 260-item ophthalmology multiple-choice question set for each model configuration. The secondary outcomes included head-to-head ranking of configurations using a Bradley–Terry model applied to paired win/loss comparisons of answer accuracy, and evaluation of generated natural language rationales using a reference-anchored, pairwise LLM-as-a-judge framework. Additional analyses assessed the accuracy-cost trade-off by calculating mean per-question cost from token usage and identifying Pareto-efficient configurations.

**Results:**

The configuration GPT-5-high achieved the highest accuracy (0.965; 95% confidence interval [CI], 0.942–0.985), significantly outperforming all GPT-5-nano variants (*P* < 0.001), o1-high (*P* = 0.04), and GPT-4o (*P* < 0.001), but not o3-high (0.958; 95% CI, 0.931–0.981). The configuration GPT-5-high ranked first in accuracy (1.66x stronger than o3-high) and rationale quality (1.11x stronger than o3-high), as judged by a reference-anchored LLM-as-a-judge autograder. Cost-accuracy analysis identified multiple GPT-5 configurations on the Pareto frontier, with GPT-5-mini-low providing the most optimal low-cost, high-performance configuration.

**Conclusions:**

This study benchmarks the GPT-5 series on a high-quality ophthalmology question-answering data set, demonstrating that GPT-5 with high reasoning effort achieved near-perfect accuracy and outperformed prior reasoning LLMs. This study also introduces an autograder framework for scalable, automated evaluation of LLM-generated answers against reference standards in ophthalmology.

**Financial Disclosure(s):**

Proprietary or commercial disclosure may be found in the Footnotes and Disclosures at the end of this article.

Large language models (LLMs) have emerged as powerful tools in medicine, capable of answering complex questions and assisting with clinical decision-making.^1^ In ophthalmology, these models have been extensively benchmarked on specialty-specific knowledge tasks.^2^ Many studies have evaluated LLM performance on ophthalmology board-style examinations and clinical cases, establishing baseline performance.^3^, ^4^, ^5^, ^6^, ^7^, ^8^

Numerous models have emerged since these initial evaluations.^9^ Recently, *reasoning models* (employing logical processing before generating outputs) have demonstrated improved performance in ophthalmic question answering. Srinivasan et al found that OpenAI’s o1 model outperformed earlier-generation models like Generative Pre-trained Transformer (GPT)-4o.^10^ Strong performance has also been reported with other reasoning models, including Gemini 2.0 Flash-Thinking (Google) and the open-source DeepSeek-R1.^4^^,^^11^

In August 2025, OpenAI released GPT-5, which is a reasoning model intended to represent the state of the art and establish a new performance frontier.^12^ Accordingly, each new generation of models warrants reassessment to determine whether improvements are sustained and to identify any potential trade-offs, including new failure modes. Ideal evaluations should use rigorously curated data sets that minimize risk of training data contamination.^13^ Prior evaluations using a closed-access sample of the American Academy of Ophthalmology’s Basic and Clinical Science Course (Basic Clinical Science Course [BCSC]) data set benchmarked GPT-3.5 at 55.8% accuracy and GPT-4 at 75.8%.^3^

We assessed the performance of GPT-5 models on the BCSC data set. We tested all 3 GPT-5 model variants (spanning different sizes/cost tiers) across 4 reasoning effort settings. The primary outcome was overall response accuracy of each configuration. The secondary outcomes included head-to-head comparisons of model configurations, a qualitative comparison of LLM responses with the BCSC ground truth, and a cost analysis of model usage.

## Methods

### The BCSC Data Set

This cross-sectional study was conducted in accordance with the Strengthening the Reporting of Observational Studies in Epidemiology guidelines and the transparent reporting of a multivariable model for individual prognosis or diagnosis LLM. In August 2025, we obtained written permission from the American Academy of Ophthalmology to use 260 questions sampled in 2023 from the 4458-question pool in the BCSC Self-Assessment Program.^3^^,^^7^ Because the data set is paywalled and publicly inaccessible, it enables consistent, closed-access benchmarking for LLM performance. The data set contains 20 text-only questions from each of the 13 ophthalmology subspecialties, as defined by the BCSC. Data set characteristics, including distribution by examination section, cognitive level, and difficulty, have been described previously.^7^ Briefly, questions were labeled as low cognitive level if they primarily assessed factual recall and high cognitive level if they required data interpretation or patient management. Difficulty was defined using the percentage of correct responses by human test-takers.

### GPT-5 Series of Models

Generative Pre-trained Transformer-5 is a series of LLMs released by OpenAI on August 7, 2025.^12^ Compared with GPT-4 and other OpenAI models, GPT-5 demonstrated superior performance on academic and human-evaluated benchmarks, including those in health.^14^ In evaluations using HealthBench,^15^ GPT-5 models outperformed GPT-4o, o1, o3, and o4-mini with higher accuracy and fewer hallucinations.^14^ Three GPT-5 models are available for testing in the application programming interface (API): GPT-5 (best model), GPT-5-mini (faster and more cost-efficient), and GPT-5-nano (fastest and most cost-efficient).^16^ OpenAI released GPT-5 as a unified system where queries are processed by the main model, with more complex tasks automatically routed to a higher-reasoning, “thinking” version of that model. The choice between main and thinking versions is made by a real-time routing mechanism based on query complexity. In the API, we were provided direct access to the thinking model. To establish a baseline, we tested OpenAI o1 and o3 with high reasoning effort. We also tested GPT-4o with a temperature of 0.3.

### Adjusting GPT-5’s Reasoning Effort

The API allows adjustment of “reasoning effort,” a parameter that controls the generation of reasoning tokens, which are internal tokens used by the model for processing and planning, before producing the final output. Higher effort settings generally result in more reasoning tokens and longer inference times but may improve performance on challenging tasks. The effort can be set to: minimal, low, medium, or high. The optimal GPT-5 reasoning effort for medical question answering has not been defined. We, therefore, tested GPT-5 on all 4 reasoning effort settings. For ease of reference, we refer to the 3 GPT-5 models with different reasoning efforts as “configurations” and label them, for example, as GPT-5-low, GPT-5-mini-medium, or GPT-5-nano-high.

### Prompting Strategy

Questions were preserved in their original multiple-choice format of 1 correct option and 3 distractors. Zero-shot prompting was used consistent with our prior work, as this approach most closely mirrors how humans answer test questions.^3^ For each question, the model received a system instruction enforcing a structured output and brevity of justification, and the question text as the user message. The system instruction is shown in Supplement 2, available at https://www.ophthalmologyscience.org.

We varied the *reasoning_effort* parameter across the 4 levels and used the API defaults. The letter <answer> was compared to the ground truth provided by the BCSC. The <rationale> natural language justification was used for secondary analyses. Token usage (input, output, and reasoning) were logged for cost analyses.

### Multiple-Choice Accuracy Evaluations

The primary outcome was to determine the accuracy of the different model configurations on the BCSC data set. For each model configuration, results were based on a single pass, given our earlier findings with GPT-3.5 demonstrating substantial to almost perfect repeatability.^3^ Before conducting primary analyses, we evaluated all 12 GPT-5 configurations (3 models × 4 reasoning efforts) on 10% of the dataset (2 questions per section, total of 26 questions) as a preliminary screening step to exclude the lowest-performing configurations. All 3 GPT-5 models with minimal reasoning effort performed the worst and were excluded from further analyses (Supplemental Table 1, available at https://www.ophthalmologyscience.org).

### Multiple-Choice Ranking Evaluations

The secondary outcomes included head-to-head comparisons of model configurations. We implemented a head-to-head LLM “arena” to perform paired comparisons for each question and derive a global ranking. For each question, every model configuration produced a letter response marked correct or incorrect. We converted these outcomes into paired comparisons between configurations: a “win” occurred when one configuration was correct and the other incorrect. For each pair of model configurations, we report a head-to-head win rate.

We then fit a Bradley–Terry (BT) model, which assigns each configuration a positive skill parameter reflecting how often it tends to win against others on discordant items.^17^ We report a BT skill metric which represents the configuration’s share of the total “ability” in the pool. Relative differences between skill values indicate how much stronger one configuration compares with another in head-to-head performance. For example, if one configuration has a skill of 0.30 and another has 0.15, the first is about twice as likely to produce a correct answer in direct comparisons.

### Autograder Evaluation Using LLM-as-a-Judge

The secondary outcomes also included an evaluation of natural language justifications (<rationale>) generated by the different model configurations. We employed a reference-anchored, pairwise judging framework using an autograder (LLM-as-a-judge). This scalable approach reduces the labor required for human grading and mitigates potential human bias.^18^

The judge model was provided with the question for context, the ground truth written by BCSC experts, and pairs of masked rationales generated by the different model configurations. To build the autograder, we used o4-mini with a custom prompt adapted from OpenAI’s “Model Scorer” from the “Evaluations” dashboard. The autograder was instructed to identify salient facts from the BCSC reference, compare each pair of rationales, and select a winner or declare a tie. The detailed prompt is depicted in Supplement 2.

After identifying the configurations with the highest multiple-choice accuracy, we selected the best-performing GPT-5 configuration (GPT-5-high) along with o1-high, o3-high, and GPT-4o to represent different model generations. For every question, 6 pairwise comparisons were performed for a total of 1560. We randomized A/B assignment per pair to mitigate position bias.^19^ Similar to the accuracy-based ranking, we report a head-to-head win rate and fit a BT model to estimate each configuration’s “skill,” then rank configurations from highest to lowest based on these values.

### Cost-Accuracy Analysis

To determine the most beneficial configurations, we plotted configuration-level accuracy against mean cost per question. We measured mean token usage per configuration (input and output tokens), converting these into a mean cost per question using OpenAI’s per-million-token pricing.^20^ We plotted accuracy against cost for each configuration and identified the Pareto-efficient configurations. A configuration was considered Pareto-efficient if there was no other configuration with both higher accuracy and lower cost. The set of such nondominated points forms the Pareto frontier. To contextualize the cost-accuracy trade-offs of the novel GPT-5 models within the broader LLM ecosystem, we extended our analysis to include leading reasoning models from Google and Anthropic—specifically Gemini 2.5 Pro, Gemini 2.5 Flash, and Gemini 2.5 Flash-Lite, as well as Claude Sonnet 4.5, Claude Haiku 4.5, and Claude Opus 4.1—thereby encompassing a full range of model sizes. Using the predefined system instruction, all Gemini models were run with dynamic reasoning enabled, allowing the model to determine autonomously when and how many reasoning tokens to allocate. Claude models were run with a fixed temperature of 1, as required to activate reasoning, and a generous reasoning budget of 4999 tokens per question.

### Statistical Analysis

For the primary outcome, accuracy was compared across configurations using Cochran Q test. Post hoc pairwise comparisons between best-performing configuration and all others were conducted with McNemar exact test, applying Holm correction for multiple comparisons. For the secondary analyses, we calculated win rates and visualized results in heatmaps, where values >0.50 indicate the row model won more often and values <0.50 indicate it lost more often. Bradley–Terry models were fitted to estimate relative skill, and configurations were ranked by descending BT skill values. Confidence intervals (CIs) for accuracy and BT skill metrics were estimated via bootstrapping with 1000 iterations. Accuracy-cost scatterplots were generated with mean cost per question plotted on a base-10 logarithmic scale to accommodate the large dynamic range in costs across configurations. All analyses were conducted in Python, version 3.13.5. Statistical significance was set at *P* < 0.05.

## Results

### GPT-5-High Was the Most Accurate

Generative Pre-trained Transformer-5-high achieved the highest accuracy (0.965; 95% CI, 0.942–0.985), whereas GPT-5-nano-low had the lowest (0.773; 95% CI, 0.723–0.823). Older generation reasoning models achieved high scores: o1 scored 0.927 (95% CI, 0.888–0.958) and o3 achieved 0.958 (95% CI, 0.931–0.981). Accuracy differed significantly across configurations (Q = 228.56; *P* < 0.001). In pairwise comparisons, GPT-5-high outperformed all GPT-5-nano configurations (*P* < 0.001), as well as o1 (*P* = 0.04), and GPT-4o (*P* < 0.001), but not o3 (*P* = 0.87). The results are summarized in Table 1. We provide the accuracy per cognitive level and question difficulty in Supplemental Tables 2-3 (available at https://www.ophthalmologyscience.org).

**Table 1:** Accuracy of Different Model Configurations at Varying Reasoning Efforts

Model	Reasoning Effort		
	Low	Medium	High
GPT-5	0.950 [0.923–0.973]	0.954 [0.927–0.977]	**0.965 [0.942–0.985]**
GPT-5-mini	0.927 [0.896–0.958]	0.942 [0.911–0.969]	0.942 [0.912–0.969]
GPT-5-nano	0.773 [0.723–0.823]	0.823 [0.781–0.865]	0.831 [0.785–0.873]
o1			0.927 [0.888–0.958]
o3			0.958 [0.931–0.981]
GPT-4o	0.865 [0.823–0.904]		

GPT = Generative Pre-trained Transformer.

The highest accuracy is in bold.

### GPT-5-High Ranked First in Head-to-Head Accuracy Comparisons

**Figure 1:**
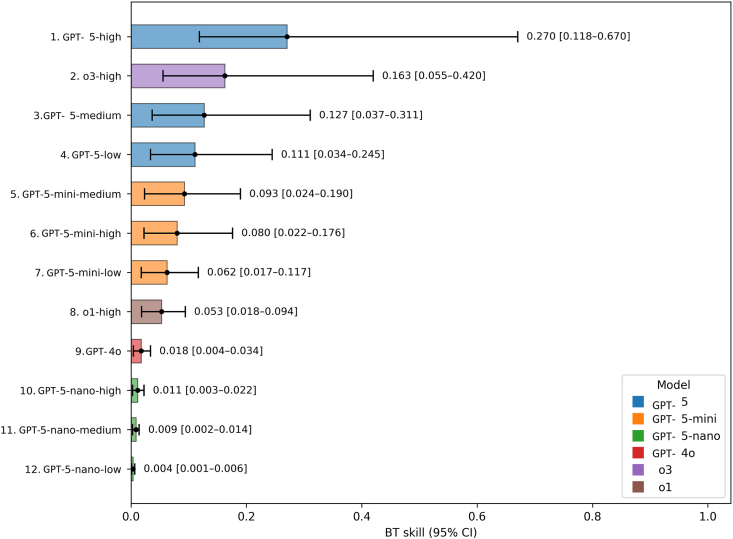
Bradley–Terry estimated accuracy skills for all model configurations ranked from highest to lowest skill. BT = Bradley–Terry; CI = confidence interval; GPT = Generative Pre-trained Transformer.

Supplemental Figure 1 (available at https://www.ophthalmologyscience.org) shows head-to-head accuracy win rates between model configurations. Overall rankings as determined by BT modeling are shown in Figure 1. The top configuration was GPT-5-high (0.270; 95% CI, 0.118–0.670), followed by o3-high (0.163; 95% CI, 0.055–0.420) and GPT-5-medium (0.127; 95% CI, 0.037–0.311). Generative Pre-trained Transformer-5 nano models ranked last. Top-ranked GPT-5-high was approximately 1.66 times stronger than o3-high and 5.10 times stronger than o1-high. We provide accuracy win rate between models per cognitive level and question difficulty in Supplemental Figures 2 and 3 (available at https://www.ophthalmologyscience.org).

### GPT-5-High Provided the Best Rationales

**Figure 2:**
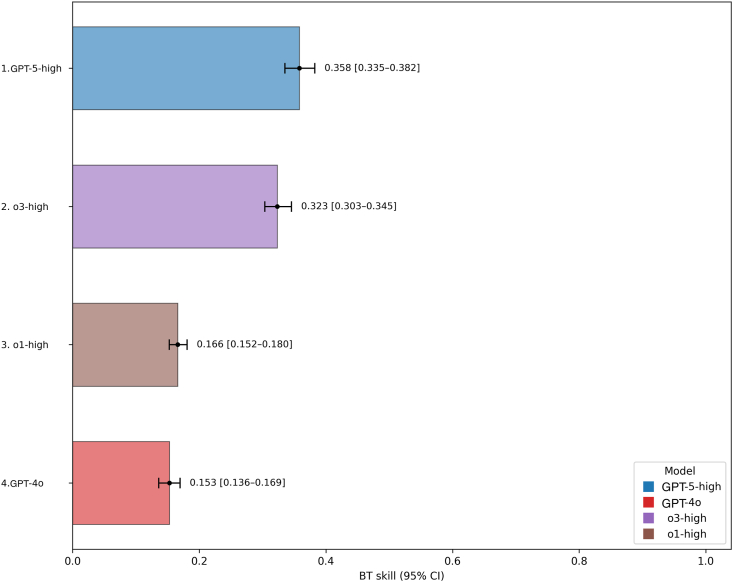
Bradley–Terry estimated skills for rationale for all model configurations ranked from highest to lowest skill. BT = Bradley–Terry; CI = confidence interval; GPT = Generative Pre-trained Transformer.

Supplemental Figure 4 (available at https://www.ophthalmologyscience.org) shows head-to-head rationale win rates between the selected model configurations. Overall rankings for rationale skill as determined by BT modeling are shown in Figure 2. Supplemental Table 4 (available at https://www.ophthalmologyscience.org) shows specific examples of a GPT-5-high win over GPT-4o and an instance of a tie. The top configuration was GPT-5-high (0.358; 95% CI, 0.335–0.382), followed by o3-high (0.323; 95% CI, 0.303–0.345), o1-high (0.166; 95% CI, 0.152–0.180), and GPT-4o (0.153; 95% CI, 0.136–0.0.169). Top-ranked GPT-5-high was estimated to be marginally (1.11 times) stronger than o3-high, 2.16 times stronger than o1-high, and 2.34 times stronger than GPT-4o. We explain win rates between models per cognitive level and question difficulty in Supplemental Figures 5 and 6 (available at https://www.ophthalmologyscience.org).

### GPT-5 Series Models Gives the Best Cost-Accuracy Trade-Offs

**Figure 3:**
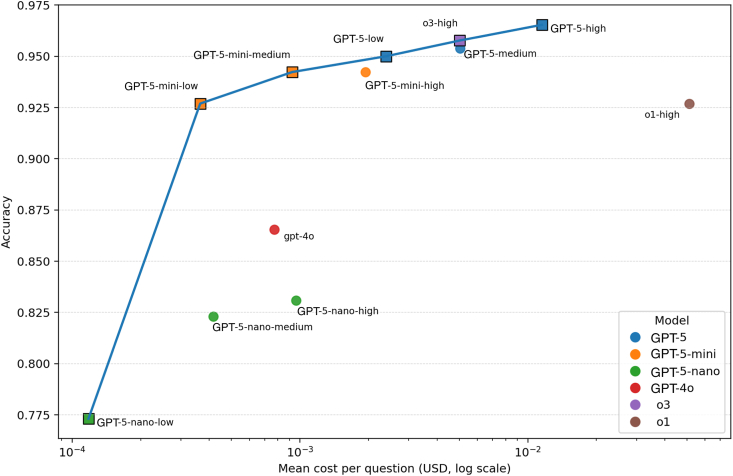
Accuracy-cost trade-off across model configurations. The x-axis is the mean cost per question (United States Dollar [USD], log scale) and the y-axis is accuracy. Square-marked configurations are Pareto-efficient, meaning no other configurations are both cheaper and more accurate. The line connects the Pareto frontier from lowest to highest cost. GPT = Generative Pre-trained Transformer.

Across all configurations, the accuracy-cost Pareto frontier was formed by GPT-5-nano-low at the ultralow-cost end and GPT-5-high at the high-cost end. Generative Pre-trained Transformer-5-mini-low was the Pareto-optimal configuration with maximal accuracy and minimal cost. Generative Pre-trained Transformer-5-medium sits near o3-high on the frontier with a similar cost-accuracy point. In contrast, o1-high costs more while delivering lower accuracy than cheaper frontier options. Figure 3 shows the accuracy-cost points for all configurations. Detailed token usage and cost calculations are shown in Supplemental Table 5 (available at https://www.ophthalmologyscience.org). In the current LLM ecosystem, as shown in Supplemental Figure 7 (available at https://www.ophthalmologyscience.org), GPT-5-nano models perform poorly and are dominated by the Gemini models, all of which lie on the Pareto frontier. Generative Pre-trained Transformer-5-mini configurations remain competitive, offering a more favorable cost–accuracy trade-off than Claude models. Generative Pre-trained Transformer-5-high maintains the highest accuracy without being the most expensive.

## Discussion

Large language models are being considered for high-stakes medicine use, yet direct comparisons of accuracy, reasoning quality, and cost-efficiency remain scarce. We evaluated the novel GPT-5 series LLMs on ophthalmic question answering. Generative Pre-trained Transformer-5 model with high reasoning effort achieved near-perfect accuracy, ranked first in head-to-head comparisons, and produced the highest-quality rationales. Cost-accuracy analyses identified GPT-5-mini with low reasoning effort as Pareto-optimal, delivering strong accuracy at minimal cost. These findings provide practical benchmarks for deploying reasoning LLMs in routine clinical ophthalmology applications.

Preliminary analyses showed that GPT-5 configurations with minimal reasoning effort consistently underperformed and produced no reasoning tokens, likely due to automated routing to the nonthinking variant; thus, these models were excluded from further analyses. In the full evaluation of the remaining 9 GPT-5 configurations alongside 3 baselines, GPT-5 with high reasoning effort achieved the highest accuracy (0.965; 95% CI, 0.942–0.985), outperforming o1 and GPT-4o, but not o3. This near-perfect performance represents a substantial improvement from GPT-3.5 (55.8%) and GPT-4 (75.8%), both tested on the same data set.^3^ Our findings align with recent reports of GPT-5 outperforming GPT-4o in reasoning and understanding scores on general medicine benchmarks.^21^ This technical achievement may be partly attributed to a broader and more recent corpus, enhanced data curation, improved reasoning through reinforcement learning, and advanced alignment tuning.

To compare configurations beyond simple accuracy, we used the BT model, which estimates the relative “skill” of each configuration from head-to-head outcomes, similar to Elo ratings in chess. This provides a more nuanced measure than raw accuracy by quantifying how often one model outperforms another.^22^, ^23^, ^24^, ^25^ In addition to testing answer accuracy, we ranked models on the quality of their generated rationales using a reference-anchored, pairwise evaluation in which an LLM-as-a-judge selected the better answer. This approach is far less labor-intensive than large-scale human grading, offering objectivity, scalability, and consistency.^26^ To our knowledge, such autograding has not been applied in ophthalmology research, which has typically relied on text-similarity metrics (e.g., Recall-Oriented Understudy for Gisting Evaluation [ROUGE] and Metric for Evaluation of Translation with Explicit Ordering [METEOR]) that may miss conceptual alignment and clinical nuance.^10^^,^^11^

In head-to-head comparisons, GPT-5 with high reasoning effort ranked first for both answer accuracy and rationale quality. For accuracy, it was approximately 1.66 times stronger than o3-high and 5.10 times stronger than o1-high. For rationale quality, it was only 1.11 times stronger than o3-high but 2.16 times stronger than o1-high, and 2.34 times stronger than GPT-4o. These findings are impressive given o1’s prior strong performance in ophthalmology.^4^^,^^11^ Our previous work found that o1 achieved the highest accuracy (0.877) on large, open-access datasets such as MedMCQA, outperforming nonreasoning OpenAI models as well as Llama 3-8B (Meta) and Gemini 1.5 Pro (Google).^10^ It also achieved the highest accuracy (0.882) on curated combinations of datasets such as the BEnchmarking LLMs for Ophthalmology compared to DeepSeek-R1 and other models.^13^

Generative Pre-trained Transformer-5 with high reasoning effort consistently outperformed all other models in both accuracy and rationale quality across question cognitive levels and difficulties. We present an example of a high-cognitive-level question from the cornea section on the management of uveal prolapse during open-globe repair (Supplemental Table 4). The ground truth specifies that uveal tissue should be reposited, and only necrotic or contaminated tissue excised. Generative Pre-trained Transformer-5 with high reasoning effort accurately determined when to reposition versus resect tissue, whereas GPT-4o prematurely recommended resection. The autograder correctly awarded the win to GPT-5. Such complex clinical cases may benefit from models with advanced reasoning capabilities that generate nuanced, context-aware responses.

Cost-accuracy analysis demonstrated that, among OpenAI models, GPT-5 series models offered the most favorable trade-offs, with the Pareto frontier spanning from GPT-5-nano-low at the ultralow-cost end to GPT-5-high at the high-accuracy end. The configuration o1-high was both more expensive and less accurate than cheaper frontier options. Generative Pre-trained Transformer-5-mini-low was the Pareto-optimal configuration, combining maximal accuracy with minimal cost. These trade-offs have practical implications for building medical applications. Configuration selection should align with the intended use case: high-effort configurations such as GPT-5-high are best suited for complex reasoning tasks like diagnostic support, where maximal accuracy justifies longer inference time and cost. In contrast, lighter variants such as GPT-5-mini-low can deliver adequate performance for high-throughput or latency-sensitive settings, including triage interfaces and patient chatbots. Model and configuration selection should be viewed within the broader LLM ecosystem spanning multiple vendors. In our exploratory analysis, Gemini models (Google) provided better cost-accuracy trade-offs than most GPT-5 models. In contrast, Claude models (Anthropic) did not provide an advantage for our use case. We plan to systematically evaluate these models and conduct cross-vendor comparisons in future work to identify the best configurations in the current ecosystem.

Understanding these trade-offs requires examining the technical factors that influence performance differences across configurations. In this study, both model size and reasoning effort influenced accuracy, but in distinct ways. Figure 3 shows that model choice is the dominant determinant of performance, with accuracy clustering by model size: GPT-5-nano performed much lower than GPT-5-mini, which in turn trailed the full GPT-5 model. Within each model, increasing reasoning effort yielded smaller but consistent improvements in accuracy. Supplemental Figure 8 (available at https://www.ophthalmologyscience.org) shows that increasing reasoning effort (demonstrated by increasing median reasoning tokens) within a model improves accuracy but with diminishing returns, meaning that most of the gain occurs between lower effort settings and later increases in effort require many more reasoning tokens to achieve only marginal additional accuracy, especially for larger models that are already near ceiling. These findings suggest that model selection should first prioritize architecture and scale, as larger models consistently outperform smaller ones even at lower reasoning effort. Once the appropriate model size is chosen, reasoning effort can be adjusted to balance accuracy, speed, and cost.

This study has limitations. Although the multiple-choice format does not fully reflect clinical decision-making, our objective was to identify optimal model configurations using a closed dataset to enable comparison with prior work. Because such evaluations rely on repeated testing with the same questions, mitigating the risk of data leakage is critical. Repeated use of the same inputs may lead to inadvertent inclusion of these questions in model training data, either through our or others’ prior use of the BCSC data set. Updated models could later be tested on material it had already encountered which would lead to spuriously elevated performance metrics. In our original ChatGPT study, we mitigated this by requesting data deletion from OpenAI and obtaining written confirmation of exclusion from model training.^7^ In our GPT-4 study, we used the API which does not contribute user inputs into training.^3^ Future work will evaluate the best-performing configurations identified in this study on more complex free-response ophthalmology cases and compare their performance with prior studies.^4^, ^5^, ^6^ It will also extend benchmarking to broader, open-access data sets such as those included in the BEnchmarking LLMs for Ophthalmology framework to assess generalizability and robustness.

Second, to our knowledge, this is the first ophthalmology study to assess rationale quality using an LLM-as-judge. This method carries potential biases, as performance depends on choice of grading model, prompt design, and evaluation format. We mitigated verbosity bias by using single-sentence justifications and position bias by randomizing A/B assignment per pair. Future work could explore longer-form rationales within this autograding framework. Finally, cost analysis was based on token usage and pricing at the time of testing; changes in API pricing, model efficiency, routing behavior, or data set characteristics could alter the relative cost-accuracy trade-offs observed here. Performance and cost may also differ on open-ended, image-based, or multistep clinical scenarios.

In conclusion, GPT-5 with high reasoning effort achieved the highest accuracy and rationale quality, outperforming most prior-generation reasoning models while offering favorable cost-accuracy trade-offs. Pareto analysis demonstrated that users can achieve high accuracy at low cost with GPT-5-mini models. These findings provide a practical framework for selecting LLM configurations in ophthalmology, whether for educational tools, research applications, or early-stage clinical decision-support systems. Future work should validate these results on complex, free-response, and multimodal ophthalmology tasks, explore the generalizability of LLM-as-a-judge for rationale evaluation, and assess costs of applied deployment.
