# Supplementary material for: Performance of GPT-5 Frontier Models in Ophthalmology Question Answering
Source: Ophthalmol Sci. 2025 Dec 6;6(2):101034. doi: 10.1016/j.xops.2025.101034 (PMC12811449; doi:10.1016/j.xops.2025.101034)
Supplement: Supplemental Material [file mmc1.pdf]

Supplemental Materials

Supplemental Table 1. Comparison of GPT-5 model configurations at different reasoning efforts on the preliminary dataset (n=20). GPT-5 models with minimal reasoning effort had the lowest accuracy and were excluded from the main analyses.

Model	Reasoning effort			
	Minimal	Low	Medium	High
GPT-5	0.846 [0.692–0.962]	1.000 [1.000–1]	0.962 [0.885–1]	0.923 [0.808–1]
GPT-5-mini	0.885 [0.768–1]	0.923 [0.808–1]	0.962 [0.885–1]	0.923 [0.808–1]
GPT-5-nano	0.692 [0.500–0.846]	0.923 [0.808–1]	0.885 [0.769–1]	0.885 [0.769–1]

Supplemental Table 2. Accuracy of model configurations at different cognitive levels

Cognitive Level	Model	Reasoning Effort	Accuracy [95% CI]
High	GPT-5	Low	0.915 [0.858–0.962]
		Medium	0.925 [0.877–0.972]
		High	0.943 [0.896–0.981]
	GPT-5-mini	Low	0.877 [0.811–0.934]
		Medium	0.896 [0.840–0.943]
		High	0.906 [0.849–0.953]
	GPT-5-nano	Low	0.708 [0.613–0.792]
		Medium	0.783 [0.708–0.858]
		High	0.783 [0.708–0.858]
	o1	High	0.887 [0.821–0.943]
	o3	High	0.943 [0.896–0.981]
	GPT-4o	N/A	0.811 [0.726–0.878]
Low	GPT-5	Low	0.974 [0.948–0.994]
		Medium	0.974 [0.948–0.994]
		High	0.981 [0.955–1.000]
	GPT-5-mini	Low	0.961 [0.929–0.987]
		Medium	0.974 [0.948–0.994]
		High	0.968 [0.942–0.994]
	GPT-5-nano	Low	0.818 [0.760–0.883]
		Medium	0.851 [0.792–0.909]
		High	0.864 [0.812–0.916]
	o1	High	0.955 [0.922–0.987]
	o3	High	0.968 [0.935–0.994]
	GPT-4o	N/A	0.903 [0.851–0.942]

Supplemental Table 3. Accuracy of model configurations at different difficulty categories

Difficulty Category	Model	Reasoning Effort	Accuracy [95% CI]
**Easy**	GPT-5	Low	0.667 [0.333–0.889]
		Medium	0.667 [0.333–0.889]
		High	0.778 [0.444–1.000]
	GPT-5-mini	Low	0.556 [0.222–0.889]
		Medium	0.556 [0.222–0.889]
		High	0.667 [0.333–0.889]
	GPT-5-nano	Low	0.444 [0.111–0.778]
		Medium	0.556 [0.222–0.889]
		High	0.556 [0.222–0.889]
	o1	High	0.556 [0.222–0.889]
	o3	High	0.667 [0.333–0.889]
	GPT-4o	N/A	0.556 [0.222–0.889]
**Moderate**	GPT-5	Low	1.000 [1.000–1.000]
		Medium	0.986 [0.966–1.000]
		High	0.993 [0.979–1.000]
	GPT-5-mini	Low	0.973 [0.945–0.993]
		Medium	0.993 [0.979–1.000]
		High	0.979 [0.952–1.000]
	GPT-5-nano	Low	0.849 [0.795–0.911]
		Medium	0.877 [0.815–0.925]
		High	0.904 [0.856–0.945]
	o1	High	0.979 [0.952–1.000]
	o3	High	0.986 [0.966–1.000]
	GPT-4o	N/A	0.952 [0.911–0.986]
**Difficult**	GPT-5	Low	0.905 [0.848–0.962]
		Medium	0.933 [0.886–0.981]
		High	0.943 [0.895–0.981]
	GPT-5-mini	Low	0.895 [0.829–0.952]
		Medium	0.905 [0.848–0.952]
		High	0.914 [0.857–0.962]
	GPT-5-nano	Low	0.695 [0.600–0.781]
		Medium	0.771 [0.686–0.848]
		High	0.752 [0.657–0.829]
	o1	High	0.886 [0.819–0.943]
	o3	High	0.943 [0.895–0.981]
	GPT-4o	N/A	0.771 [0.686–0.848]

Supplemental Table 4. Examples of a GPT-5-high win and tie versus GPT-4o

Question	BCSC Ground truth and model responses
What is the best way to manage uveal prolapse when it occurs during an acute open-globe repair?<br>A. reposit tissue<br>B. resect tissue<br>C. repair/reconstruct tissue<br>D. cauterize tissue	**BCSC ground truth**<br>If uvea or retina (seen as translucent, tan tissue with extremely fine vessels) protrudes, the surgeon can reposit it, using a gentle sweeping technique through a separate limbal incision, with the assistance of viscoelastic injection to keep the anterior chamber formed. Only in cases of frankly necrotic, macerated, or contaminated tissue should prolapsed uveal tissue be excised. Cauterization of uveal tissue would not be appropriate. Reconstruction of iris tissue, using techniques such as the McCannel suture, may be indicated in a follow-up surgery, but not during the repair of an acute open globe.<br><br>**GPT-5-high rationale**<br>During primary open-globe repair, viable prolapsed uveal tissue should be gently reposited, with resection reserved only for devitalized or contaminated tissue.<br><br>**GPT-4o rationale**<br>Resecting the prolapsed uveal tissue is preferred to prevent further complications and infection<br><br>**Autograder vote =** GPT-5-high
A 6-month-old child presents with constant tearing, moderate mucopurulent discharge, and a white/quiet eye. What is the most likely diagnosis?<br>A. nasolacrimal duct (NLD) obstruction<br>B. infectious conjunctivitis<br>C. dacryoadenitis<br>D. canalicular dysgenesis	**BCSC ground truth**<br>Canalicular dysgenesis leads to impaired drainage of tears, resulting in epiphora without associated signs of dacryocystitis, such as redness, swelling, pain, or mucopurulence. Infectious conjunctivitis typically causes a red eye and presents with tearing and irritation. NLD obstruction, either as a primary congenital condition or secondary to edema of the nasal tissue during an upper respiratory illness, causes tearing. Presentation can range from minimal or no discharge to copious discharge and associated dacryocystitis. Dacryoadenitis is inflammation of the lacrimal gland.<br><br>**GPT-5 high rationale**<br>A white, quiet eye with constant tearing and mucopurulent discharge in an infant is classic for nasolacrimal duct obstruction rather than conjunctivitis or lacrimal gland pathology.<br><br>**GPT-4o rationale**<br>The symptoms of constant tearing and mucopurulent discharge with a white/quiet eye are characteristic of nasolacrimal duct obstruction in infants.<br><br>**Autograder vote =** tie

Supplemental Table 5. Token usage and cost calculations of model configurations at different reasoning efforts. For tokens, median and interquartile range [IQR] are shown to reflect typical reasoning efforts length under heavy-tailed distributions. *Reasoning tokens are included in output tokens

Model	Reasoning Effort	Accuracy	Mean Cost Per Question (USD)	Median [IQR] Input Tokens	Median [IQR] Output Tokens	Median [IQR] Reasoning Tokens*	Median [IQR] Total Tokens
GPT-5	Low	0.950	0.002	123 [112–143]	178 [116–248]	128 [64–192]	305 [238–393]
	Medium	0.954	0.005	123 [112–143]	362 [240–559]	320 [192–512]	485 [365–685]
	High	0.965	0.012	123 [112–143]	752 [498–1261]	704 [448–1216]	877 [621–1415]
GPT-5-mini	Low	0.927	0.000	123 [112–143]	116 [109–182]	64 [64–128]	254 [225–320]
	Medium	0.942	0.001	123 [112–143]	306 [239–438]	256 [192–384]	445 [365–595]
	High	0.942	0.002	123 [112–143]	618 [444–1012]	576 [384–960]	736 [585–1143]
GPT-5-nano	Low	0.773	0.000	123 [112–143]	236 [167–310]	192 [128–256]	352 [276–457]
	Medium	0.823	0.000	123 [112–143]	741 [501–1265]	704 [448–1216]	875 [625–1394]
	High	0.831	0.001	123 [112–143]	1722 [1066–2860]	1664 [1024–2816]	1887 [1190–2972]
o1	High	0.927	0.051	123 [112–143]	546 [360–799]	512 [320–768]	681 [500–920]
o3	High	0.958	0.005	123 [112–143]	358 [239–507]	320 [192–448]	478 [372–667]
GPT-4o	N/A	0.865	0.001	123 [112–143]	44 [41–48]	0 [0–0]	167 [152–188]

Supplemental Figure 1. Head-to-head accuracy win rates between model configurations. Each cell shows the proportion of times the row model was correct and the column model was not. Values > 0.50 mean the row model won more often; values < 0.50 mean it lost more often.

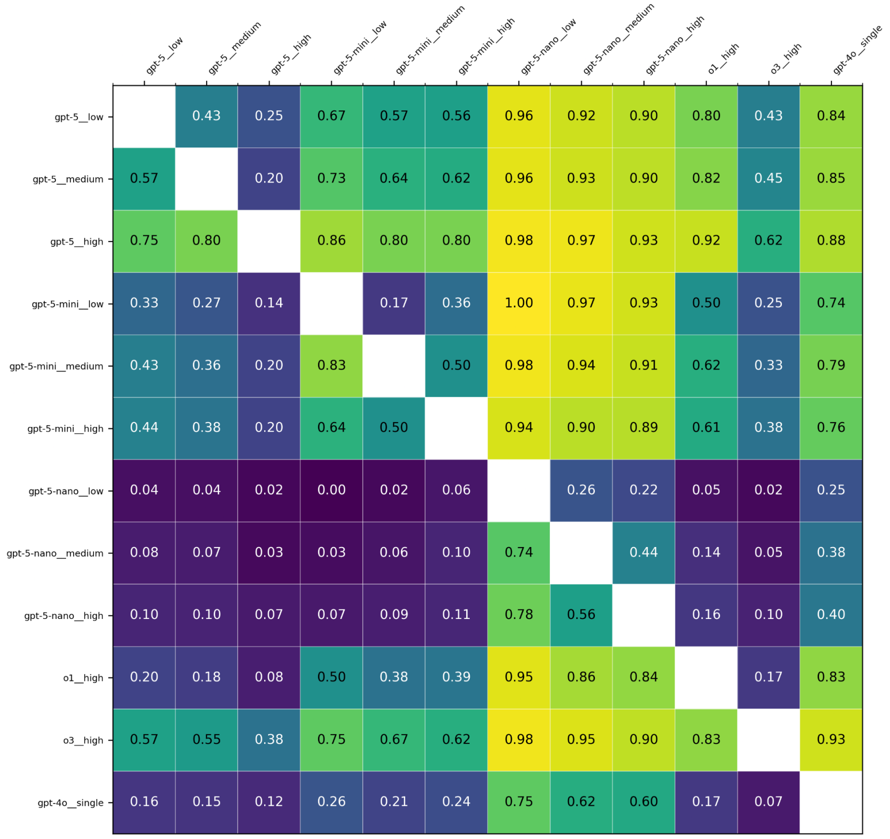

Supplemental Figure 2. Head-to-head accuracy win rates between model configurations per cognitive level. Each cell shows the proportion of times the row model was correct and the column model was not. Values > 0.50 mean the row model won more often; values < 0.50 mean it lost more often.

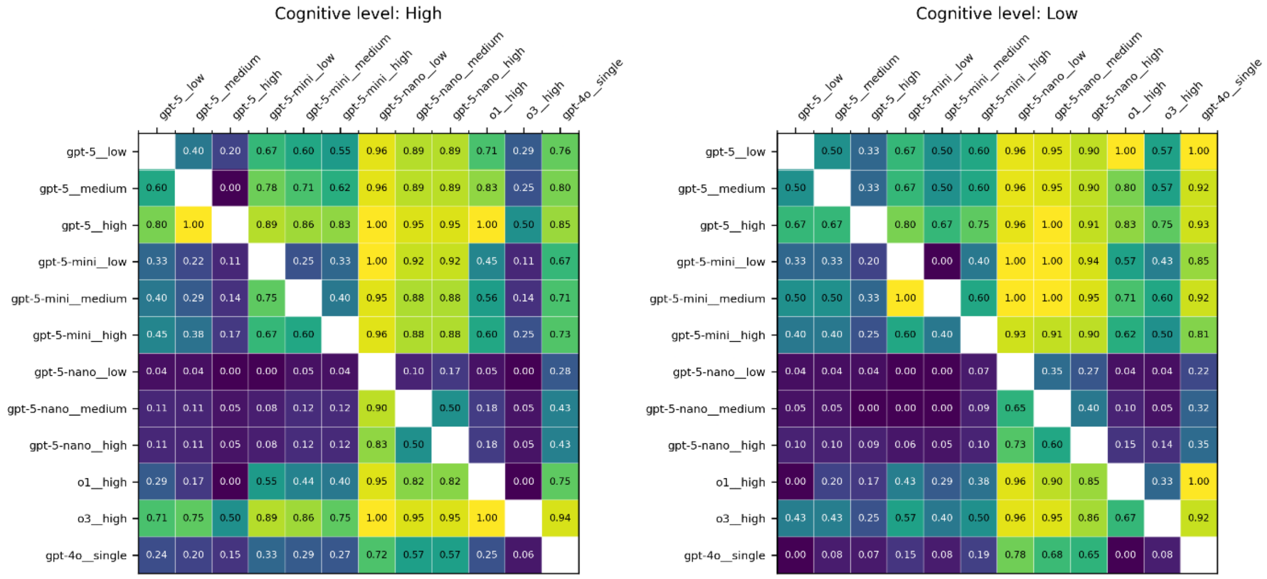

Supplemental Figure 3. Head-to-head accuracy win rates between model configurations per question difficulty. Each cell shows the proportion of times the row model was correct and the column model was not. Values > 0.50 mean the row model won more often; values < 0.50 mean it lost more often.

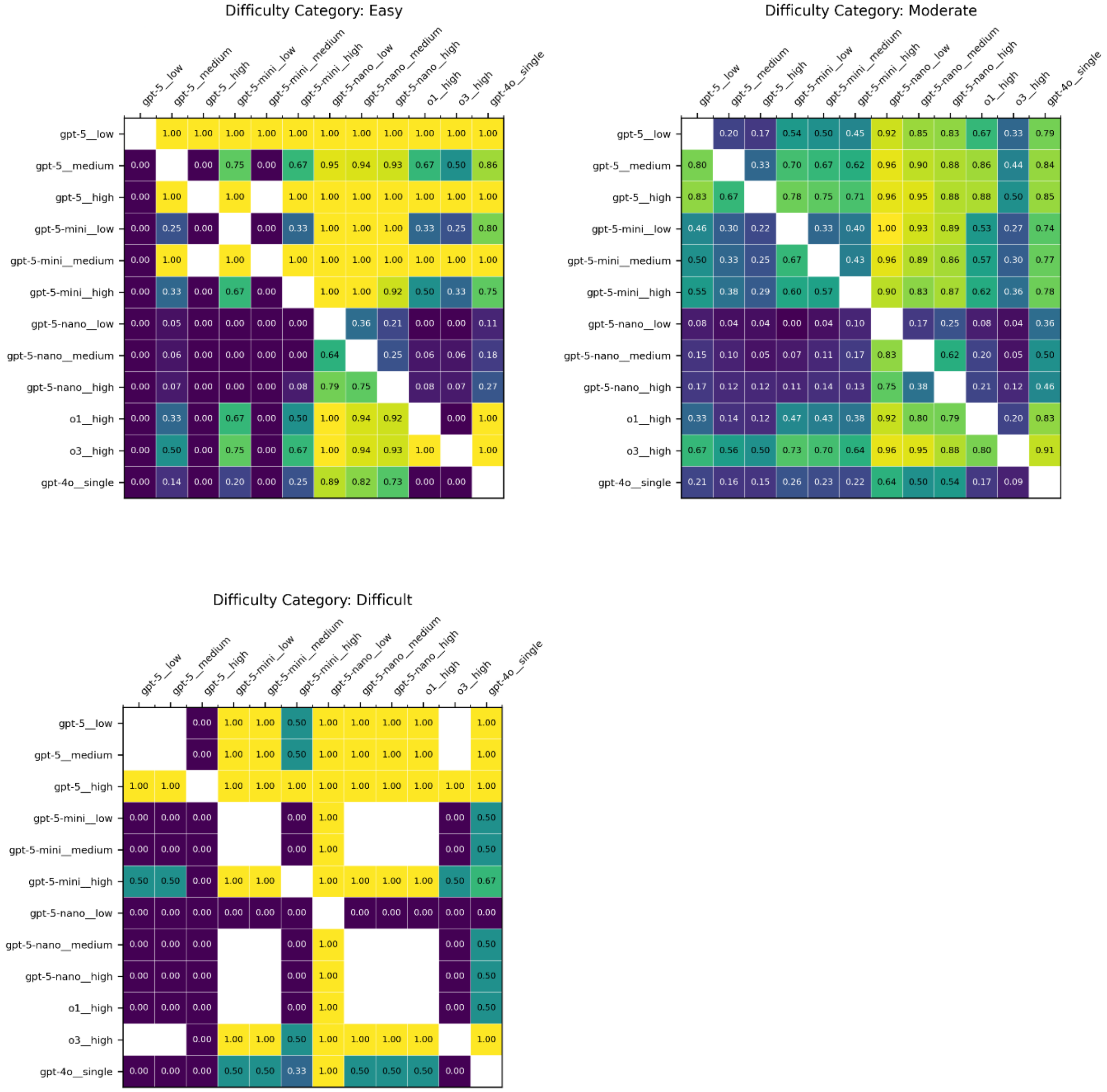

Supplemental Figure 4. Head-to-head rationale win rates between model configurations. Each cell shows the proportion of times the row model was correct and the column model was not. Values > 0.50 mean the row model won more often; values < 0.50 mean it lost more often.

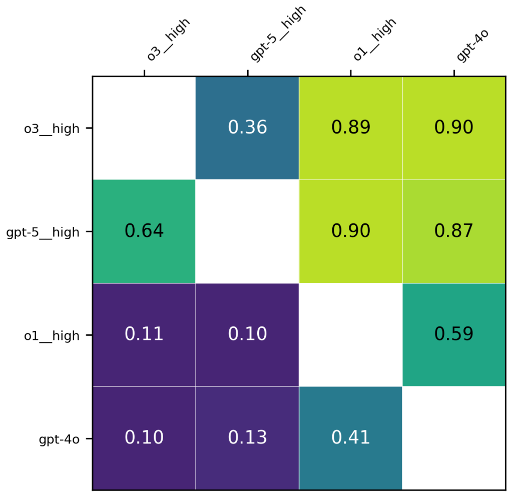

Supplemental Figure 5. Head-to-head rationale win rates between model configurations per cognitive level. Each cell shows the proportion of times the row model was correct and the column model was not. Values > 0.50 mean the row model won more often; values < 0.50 mean it lost more often.

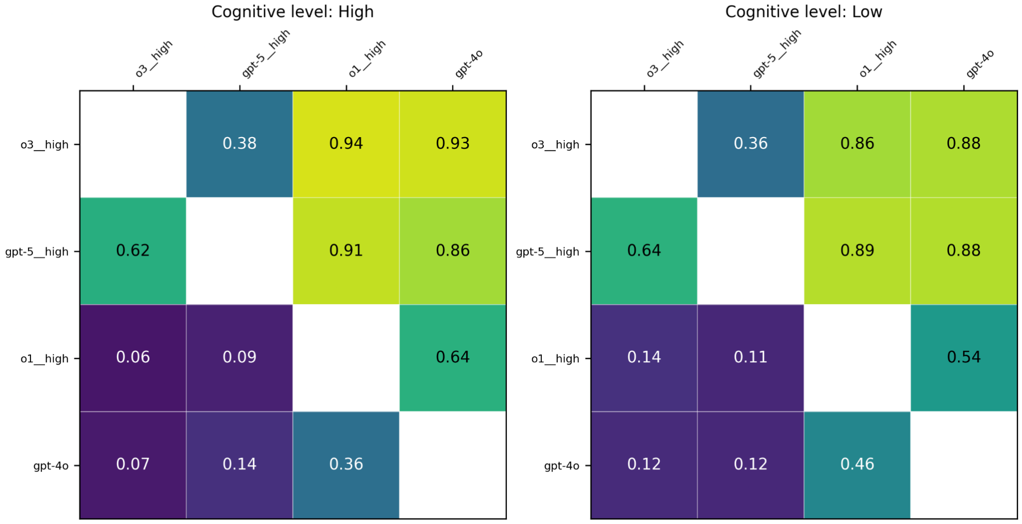

Supplemental Figure 6. Head-to-head rationale win rates between model configurations per question difficulty. Each cell shows the proportion of times the row model was correct and the column model was not. Values > 0.50 mean the row model won more often; values < 0.50 mean it lost more often.

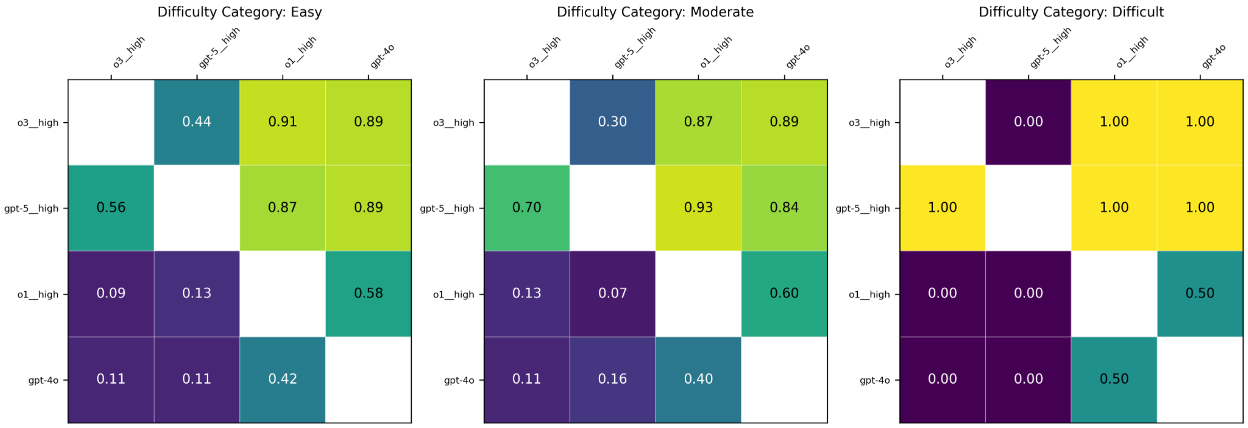

Supplemental Figure 7. Accuracy-cost trade-off across model configurations in comparison with Gemini and Claude models. The x-axis is the mean cost per question (USD, log scale) and the y-axis is accuracy. Square marked configurations are Pareto-efficient, meaning no other configurations are both cheaper and more accurate. The line connects the Pareto frontier from lowest to highest cost.

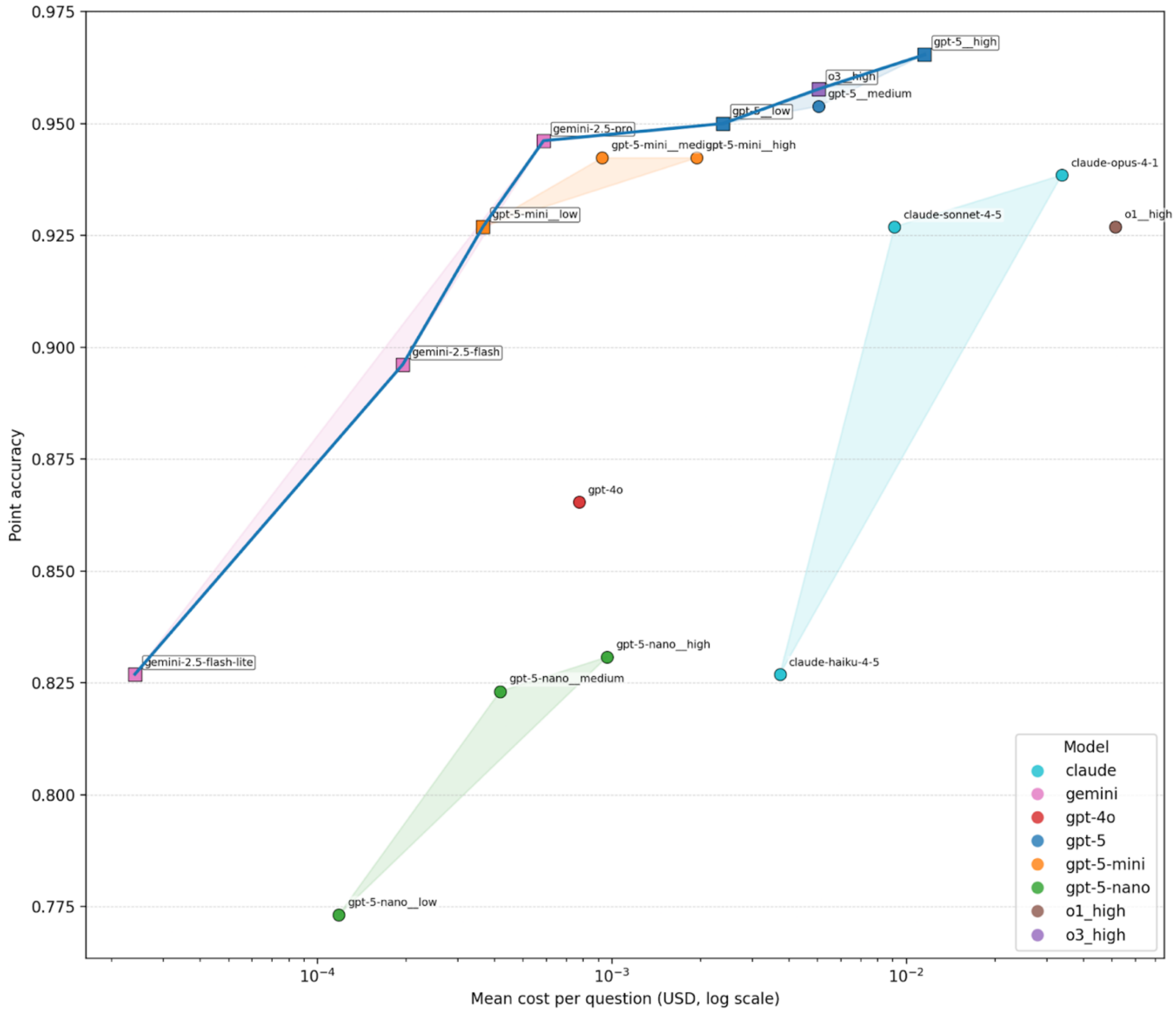

Supplemental Figure 8. Intra-model scaling of accuracy with reasoning effort. Each line represents a single model size, showing how accuracy changes as median reasoning tokens per question increase across effort settings. Steeper slopes indicate that the model gains accuracy when given more reasoning tokens. Flatter slopes indicate diminishing returns, where additional reasoning provides little improvement.

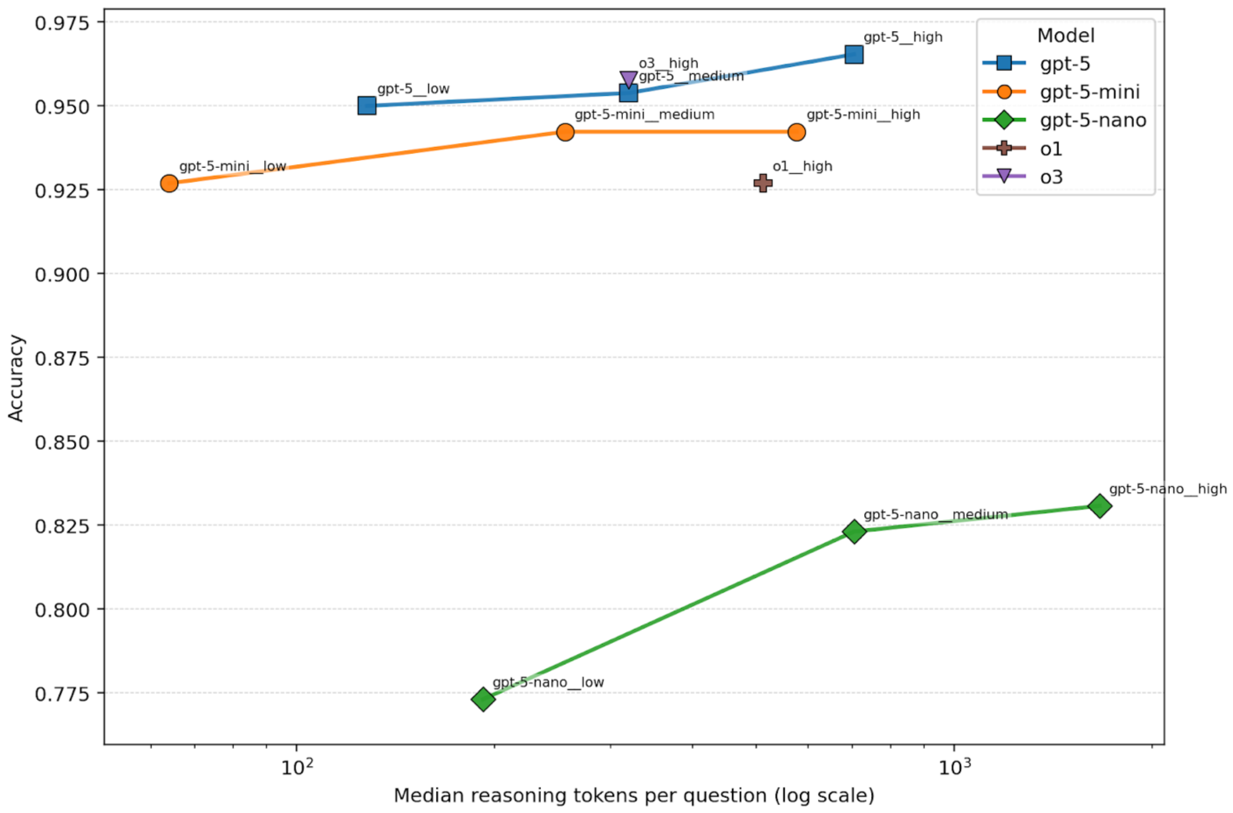

Prompting Strategy System Prompt

Answer this question. Return strict JSON with two keys: ‘answer’ and ‘rationale’. ‘answer’ MUST be a SINGLE CAPITAL LETTER (A–D) only. ‘rationale’ MUST be a ONE-SENTENCE concise justification for your answer (no step-by-step).

Autograder Evaluation Prompt

You judge two single-sentence rationales generated while answering an ophthalmology multiple-choice question against an authoritative reference. Use the QUESTION only for context; grade alignment to the REFERENCE_EXPLANATION.

QUESTION: {{question_text}}

REFERENCE_EXPLANATION: {{bcsc_reference_text}}

RATIONALE_A: {{rationale_a}}

RATIONALE_B: {{rationale_b}}

Silently extract 2–4 salient facts from the reference.

Choose the rationale that better matches those facts.

Major contradictions or wrong mechanism/pathology are fatal.

Return ONLY this one-line JSON:

{"winner":"A"|"B"|"tie"}
